# Advanced biosensing strategies for high-risk foodborne pathogens: a comprehensive review of *Salmonella* and *Listeria monocytogenes*

**DOI:** 10.1016/j.fochms.2026.100417

**Published:** 2026-05-20

**Authors:** Suzan Efife Gumush, Rima Al-Attar, Ebba Sandbecker, Santosh Pandit

**Affiliations:** a Department of Chemistry and Molecular Biology, University of Gothenburg, Gothenburg 405 30, Sweden; b LayerLogic AB, Medicinaregatan 8A, 413 90 Gothenburg, Sweden; c Systems and Synthetic Biology Division, Department of Life Sciences, Chalmers University of Technology, SE-412 96 Gothenburg, Sweden

**Keywords:** Biosensor, Food pathogens, *Listeria monocytogenes*, Salmonella

## Abstract

The goal of this review is to comprehensively analyse advances in biosensors specifically for *Listeria monocytogenes* and *Salmonella* spp., two of the leading causes of foodborne illness and mortality worldwide. Unlike previous reviews that broadly survey bioreceptors or transducer classes across many pathogens, this work provides a focused, pathogen-specific comparison, mapping current trends in bioreceptor development and transducer choices uniquely for these two high-priority pathogens. Additionally, while artificial intelligence (AI) applications in biosensing have been reviewed before, this article is the first to examine how AI-driven tools can directly accelerate biosensor design, optimisation, and decision-making for *Listeria* and *Salmonella* detection. Importantly, this review bridges the persistent gap between laboratory concepts and real-world implementation by critically evaluating which biosensing strategies have genuine potential for scalability, portability, and routine use in the food industry. Together, these elements position the review as a practical roadmap for steering future biosensor research toward field-ready, industry-relevant solutions.

## Highlights

• Biosensor technology has strong potential to advance bacterial detection in food.
• Widely used bioreceptors in biosensors are discussed with their pros and cons.
• Potential of electrochemical, optical and piezoelectric devices are discussed.
• Bioreceptors, transducer engineering, and AI-assisted optimisation are keys to advance biosensors for food born pathogens.

## 1 Introduction

Foodborne pathogens are infectious microorganisms capable of contaminating food, and their ingestion can lead to disease in humans (Abebe et al., 2020). These microorganisms can include viruses, bacteria, or parasites; however, among them, bacteria are considered the most prevalent infectious agent, causing approximately two-thirds of foodborne diseases worldwide (Abebe et al., 2020). Examples of such bacterial pathogens include *Listeria monocytogenes* and Salmonella species. While this article focuses on *Listeria monocytogenes* and Salmonella species, it does not exclude the importance of other foodborne pathogens. These two are selected because they represent complementary and highly relevant food safety scenarios in modern systems. Both are globally significant, associated with Listeriosis and Salmonellosis, combining notable incidence with substantial public health impact.

Importantly, they illustrate distinct contamination pathways: Salmonella is mainly linked to raw or minimally processed animal products and risks related to inadequate cooking or cross-contamination, whereas *Listeria monocytogenes* is associated with ready-to-eat foods, post-processing contamination, and the ability to grow under refrigeration. To begin with, *L. monocytogenes* is of particular concern because, although it has a low infection rate in the general population (approximately 0.1–11.3 cases per 100,000 people according to Singh et al., (P. Singh & Manik, 2025), the illness it causes, known as listeriosis, is often very severe. Singh et al. (P. Singh & Manik, 2025) also noted that 90% of individuals with listeriosis require hospitalization, and about 20–30% of cases can result in death. Unlike L. *monocytogenes*, Salmonella is much more common and is considered one of the major foodborne pathogens, causing a significant global health and economic burden (Lamichhane et al., 2024). Its prevalence worldwide ranges between 200 million to 1 billion cases each year, with approximately 155,000 deaths (Lamichhane et al., 2024).

Given their prevalence and severity, it is crucial to detect these bacteria rapidly, accurately, and at very low concentrations to prevent outbreaks (Y. Shen et al., 2021; P. Singh & Manik, 2025). Current detection methods include culture-based techniques, immunoassays, and molecular methods. Culture-based methods typically begin with enrichment steps, followed by the isolation of colonies, and then undergo confirmatory analysis using biochemical, morphological, or genetic tests to confirm the presence of the target pathogen (Ferone et al., 2020). These methods are regarded as the standard reference method when it comes to detecting bacteria (Ferone et al., 2020; P. Singh & Manik, 2025). However, they have some limitations, which include being very slow, taking approximately 5–7 days due to the multiple steps involved in the method (Ferone et al., 2020; Maciorowski et al., 2006). In addition, they require skilled personnel and an appropriate laboratory setting to carry out these tests (Ferone et al., 2020). Due to these limitations, they are not considered ideal for rapid detection (P. Singh & Manik, 2025).

In contrast, immunological (e.g., ELISA) and molecular methods (e.g., PCR, qPCR, and rt-PCR) are considered more rapid, sensitive, and specific (Ferone et al., 2020; P. Singh & Manik, 2025). Their sensitivity and specificity can be due either to the antibody binding to the pathogen-specific antigen in the case of immunological assays, or due to the binding to pathogen-specific DNA sequence in the case of molecular methods (Ferone et al., 2020). Despite their advantages, these methods have limitations, as they require qualified lab personnel, time consuming, costly and complex instruments, and in some cases, they may produce inaccurate results because food-derived substances can interfere with the detection test (Ferone et al., 2020; Y. Shen et al., 2021; P. Singh & Manik, 2025).

The traditional detection methods, although considered the gold standard, have limitations as discussed above (Castillo-Henríquez et al., 2020). These constraints highlight the need for faster, more efficient, and innovative approaches that reduce reliance on high-tech equipment and long processing times (L. Shen et al., 2026). This has driven the development of emerging technologies capable of detecting foodborne pathogens while prioritising high sensitivity and specificity. One such contemporary method is the biosensor (Sezgintürk, 2020). Novel innovations in biosensor technology are transforming current detection strategies by offering rapid, accurate, selective, and highly sensitive analysis (Y. Shen et al., 2021).

In this context, the present review aims to collate and critically evaluate recent developments in biosensor technologies used for the detection of L. *monocytogenes* and *Salmonella*, with a specific emphasis on how different bioreceptor classes and transducer platforms have been applied to these two pathogens. By examining the literatures in the field, this review identifies emerging patterns in assay design, highlights technological strengths and limitations across bioreceptor categories, and clarifies how detection strategies differ between *Listeria* and *Salmonella*. In addition, the review assesses the growing contribution of artificial intelligence (AI) to biosensor optimisation and data interpretation within this pathogen-specific context. Ultimately, the goal is to establish a structured overview of the field that can guide future research directions and support the development of biosensing technologies that are not only scientifically robust but also compatible with practical food industry requirements.

## 2 Introduction to biosensors

Biosensors are analytical devices capable of converting a biological response arising from the interaction between an analyte and a bioreceptor into an electrochemical, optical, or piezoelectric signal via a transducer (L. Shen et al., 2026). These devices offer numerous advantages over traditional and molecular detection methods, such as high sensitivity and specificity, rapid and real-time results, cost-effectiveness, portability, on-site usability, convenience, and versatility across different analytes. Owing to these benefits, biosensors are gaining increasing attention across the food, medical, and environmental sectors (Sezgintürk, 2020).

**Fig. 1:**
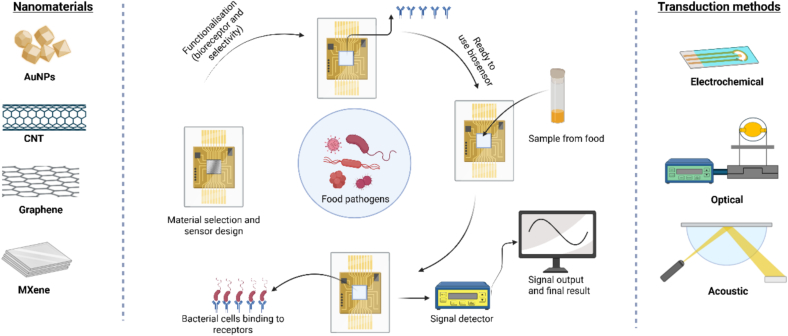
Schematic illustration of the biosensing workflow for foodborne pathogen detection. The process encompasses the functionalisation of the biosensor interface. Once the surface is functionalised, the food matrix is dropped on the biosensor surface, enabling bacterial binding to the bioreceptor. The signal detector measures the binding event to display the result. Various nanomaterials can be used for surface modifications such as graphene and Mxene. Signal transduction can occur through electrochemical, optical, or piezoelectric systems. Created with BioRender.com.

A biosensor is constructed using five main components: the analyte, bioreceptor, transducer, electronic circuitry, and the display (Naresh & Lee, 2021). In practical food safety applications, the target analyte (e.g., Salmonella or Listeria) must first be isolated or enriched from complex food matrices. Once the analyte binds to the bioreceptor, which is immobilised onto a nanomaterial-modified surface, a physiochemical change is generated (Ferone et al., 2020). This change is subsequently converted into a measurable electrical, optical, or acoustic signal by the transducer (L. Shen et al., 2026). The integrated electronics then process and amplify this raw signal, which is finally presented on a user-friendly interface or software system that visualises the analytical results in a graphical format as seen in Fig. 1.

## 3 Bioreceptors

**Fig. 2:**
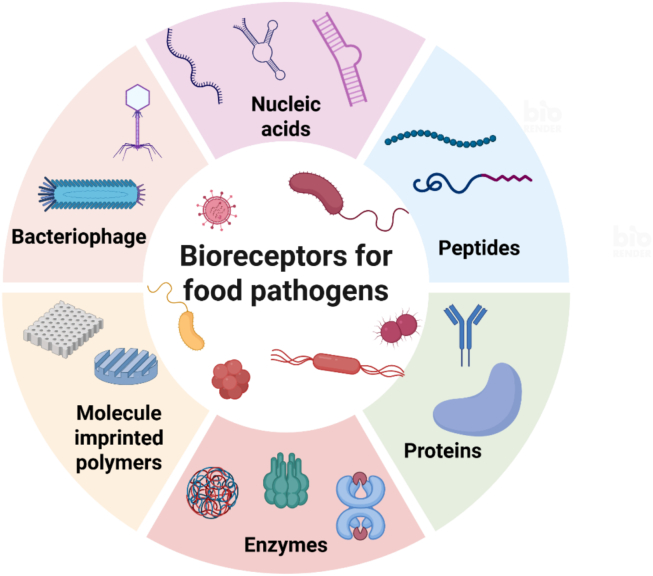
An overview of the various biorecognition elements used in biosensors for foodborne pathogen detection. Different recognition mechanisms such as enzymes, nucleic acids, peptides, bacteriophage, and molecularly imprinted polymer (MIP) recognition, are displayed to highlight the selective binding of the bioreceptors to L. *monocytogenes* and Salmonella in biosensing detection. Created with Biorender.com.

Bioreceptors constitute one of the critical components of a biosensor, as they largely determine its selectivity toward the target pathogen. They also influence the limit of detection (LOD) and broaden the scope of biosensor applications through their ability to recognise a wide range of analytes (Sezgintürk, 2020). As displayed in Fig. 2, bioreceptors involved in L. *monocytogenes* and *Salmonella* biosensing include antibodies, enzymes, nucleic acids, peptides, bacteriophages, molecularly imprinted polymers (MIPs) and whole-cells (Sezgintürk, 2020; L. Shen et al., 2026). High stability, strong binding affinity, and a robust interaction with the target are essential characteristics when selecting a bioreceptor (Sezgintürk, 2020).

Table 1 summarises the key advantages and limitations of commonly used bioreceptors. These advantages and limitations are context-dependent and may vary depending on assay conditions and target environments.

**Table 1:** Overview of the main bioreceptor types used for pathogen biosensing, with their key advantages and limitations.

Bioreceptor type	Advantages	Limitations
Antibodies (monoclonal, polyclonal, recombinant)	Typically exhibit high binding affinity (Kd ∼10^−7^–10^−11^ M) and strong specificity toward surface epitopes due to well-defined antigen–antibody interactions.	Susceptible to denaturation under non-optimal pH and temperature due to protein conformational instability; high production cost associated with biological expression systems; polyclonal antibodies may exhibit batch-to-batch variability
Enzymes (natural enzymes, nanozymes)	Natural enzymes provide substrate-specific catalytic activity enabling signal amplification; nanozymes offer enhanced chemical stability and cost-effective synthesis	Natural enzymes are sensitive to environmental conditions and prone to degradation; nanozymes lack intrinsic biological specificity and typically require coupling with selective bioreceptors
Molecularly Imprinted Polymers (MIPs)	Provide synthetic recognition sites with high chemical and thermal stability; suitable for use in complex matrices; long shelf life	Imprinting whole-bacterial cells is challenging due to their large size and structural complexity; incomplete template removal may reduce binding efficiency; often require conductive nanomaterials to improve signal transduction
Peptides (AMPs, engineered peptides)	Small size enables high surface accessibility and rapid binding; can be synthesised with relatively low cost and improved reproducibility	Generally lower specificity compared to antibodies or aptamers; susceptible to enzymatic degradation in food matrices; selection and optimisation for whole-cell targets can be complex
Nucleic Acid Probes	High sequence specificity via Watson–Crick base pairing; suitable for genosensing applications targeting pathogen DNA/RNA	Cannot directly detect intact cells; unable to distinguish viable from non-viable organisms without additional processing steps
Aptamers	Capable of high-affinity binding through sequence-dependent folding into 3D structures; smaller size enables high surface density on transducers; chemically synthesised with good reproducibility	Performance is sensitive to ionic strength and matrix composition due to structural dependence; susceptible to nuclease degradation in complex food samples; SELEX selection can be time-intensive with variable success rates
Bacteriophages	Exhibit host specificity via receptor-binding proteins, enabling selective recognition of viable bacterial cells; generally stable across a range of environmental conditions; low-cost production via bacterial hosts	Sensor performance depends on orientation and immobilisation efficiency; potential lytic activity may interfere with signal stability; storage and handling require maintenance of biological activity
Whole cells	Enable detection based on biological activity, providing signal amplification and functional sensing	Highly sensitive to environmental conditions (e.g., nutrients, temperature), leading to poor reproducibility; regulatory constraints for genetically modified systems

## 4 Antibodies

Antibodies are large Y-shaped glycoproteins widely employed as affinity biorecognition elements due to their high specificity toward target molecules (Khaleque et al., 2024). Several recognition elements are used in biosensors, including monoclonal antibodies (mAbs). These are derived from a single cell clone and bind to a single, specific epitope (Sezgintürk, 2020). They offer high binding affinity (typically in the range of nanomolar to picomolar) and strong specificity toward surface epitopes. This specificity arises from well-defined antigen–antibody interactions involving hydrogen bonding, van der Waals forces, hydrophobic interactions, and electrostatic forces (Yu et al., 2017). However, their production requires complex and costly mammalian cell culture systems followed by extensive purification steps (Chames et al., 2009; Naresh & Lee, 2021). For example, Servarayan et al. (Servarayan et al., 2023) employed a monoclonal anti-*Listeria* antibody conjugated to an imine-based fluorophore to create a simple fluorescence immunoassay, achieving highly sensitive detection with an LOD of 3.2 CFU/mL and excellent selectivity without requiring blocking steps.

Polyclonal antibodies (pAbs) on the other hand, originate from multiple cell clones and bind to multiple epitopes. They are cheaper and faster to produce, though more prone to cross-reactivity which may lead to false positive result (Naresh & Lee, 2021). Fernández Blanco et al. (Fernández Blanco et al., 2023b) evaluated both polyclonal and monoclonal anti-*Listeria* antibodies on a silicon-nitride ring resonator platform, reporting LOD values around 10^1^–10^2^ CFU/mL and clear discrimination between L. *monocytogenes* and *L. innocua*. Notably, the polyclonal antibody showed stronger immobilisation and broader binding capacity, whereas the monoclonal antibody offered higher specificity at low concentrations. Similarly, for *Salmonella* Fernández Blanco et al. (Fernández Blanco et al., 2023a). demonstrated that both monoclonal antibodies and polyclonal antibodies could be integrated into a silicon-nitride photonic biosensor, achieving an LOD of ∼10 CFU/mL and 100% sensitivity and negative predictive value in food samples.

Despite these advantages, antibodies generally exhibit high specificity toward target epitopes and strong binding affinity (Kd ∼10^−7^–10^−11^ M) (Adams et al., 2026; Wilson & Soh, 2020), but are susceptible to denaturation under non-optimal environmental conditions (e.g., temperature and pH) due to thermal unfolding and loss of conformational stability (Bailly et al., 2020). Moreover, antibodies display batch-to-batch variability, therefore resulting in the possibility of limited reproducibility (Centane & Nyokong, 2022; Nagata et al., 2025; X. Pan et al., 2023). Since antibody–analyte binding does not involve intrinsic redox reactions, transduction often depends on impedance-based detection, potentially lowering sensitivity (Naresh & Lee, 2021).

Whereas recombinant antibodies such as nanobodies are genetically engineered to enhance stability, affinity, and orientation on sensor surfaces (Zeng et al., 2012). Their smaller molecular size improves epitope access, and orientation-controlled immobilisation enhances binding efficiency (Rudenko et al., 2021; Zeng et al., 2012). However, not all target analytes can be easily engineered, limiting availability (Khaleque et al., 2024). Bai et al. (Bai et al., 2023) developed a dual-nanobody immunomagnetic ELISA platform for *S. enteritidis*, reaching an LOD of 3.2 × 10^3^ CFU/mL and enabling detection from multiple food matrices within 4 h.

## 5 Enzymes

Enzymes are biological catalysts composed of amino acids forming one or more polypeptide chains, giving rise to their highly specific catalytic properties (Khaleque et al., 2024). In biosensors, enzymes produce measurable signals through catalytic reactions with the analyte or by amplifying the response of another recognition element (Naresh & Lee, 2021).

Recent work has demonstrated a growing interest in enzyme-based nucleic acid recognition systems, most notably CRISPR-associated nucleases that reflect renewed relevance of natural enzymes in pathogen detection (Engku Abd Rahman et al., 2026). In recent work by Liu et al. (Liu et al., 2026), a visual detection platform integrating CRISPR-Cas12b with a loop-mediated isothermal amplification (LAMP) system was developed for detecting L. *monocytogenes*. By utilising the collateral cleavage activity of the Cas12b enzyme, the system serves as a signal amplifier when recognising the target *lmo0753* gene, producing a result on a lateral flow strip. An LOD of 20 CFU/g for pork samples was achieved, demonstrating a sensitive, rapid, equipment-free detection method. Similarly, Duan et al. (Duan et al., 2025) developed a portable CRISPR/Cas12a biosensor for the detection of *S. typhimurium*. In this platform, the Cas12a enzyme triggers the release of glucose oxidase (GOx), which catalyses the oxidation of glucose to produce gluconic acid, resulting in a measurable pH change. The system employed an accessible smartphone-based imaging program for signal readout, achieving an LOD of 1.41 × 10^2^ CFU/mL in chicken samples. While offering high sensitivity and specificity for pathogenic bacteria in food, a limitation of this CRISPR-based approach is its inability to distinguish between live and dead bacteria, which may lead to false positives results (Yang, Ledesma-Amaro, et al., 2023).

As biosensor technology has evolved, the field has also gradually shifted toward nanozymes, which are artificial enzyme-mimicking nanomaterials (Arshad et al., 2022). This transition reflects a broader trend in bioreceptor design. Nanozymes are used predominantly as signal amplification labels as they often lack intrinsic biological specificity (Arshad et al., 2022). Nanozymes are typically conjugated to another bioreceptor, that provides the selective binding, while the nanozyme enhances the measurable output (Altalbawy et al., 2026). Li et al. (W. Li et al., 2025) developed an electrochemical biosensor for L. *monocytogenes* in which the bioreceptors are doripenem (capture ligand) and monoclonal antibodies (detection ligand). Where attached MXene-CuBTC nanozyme acts purely as a catalytic label that enhances the electrochemical signal, enabling an LOD of 1.9 × 10^1^ CFU/mL. In another work by Deng et al. (Deng et al., 2026), a mannose-functionalised manganese Prussian blue nanozyme (Man-MPN) was utilised in a lateral flow immunoassay (LFIA) platform for *S. typhimurium*. In this system, mannose functions as the bioreceptor while the nanozyme provides oxidase-like catalytic amplification, improving the visual LOD to 10^3^ CFU/mL.

While the advantages of natural enzymes include high specificity and dependable output signal in relation to analyte quantification (Naresh & Lee, 2021), they suffer from various limitations such as short operational lifetimes, potential instability and denaturation due to changes in the environmental or protease digestion, which can limit catalytic functions and long-term sensor performance (Khaleque et al., 2024; Shamsabadi et al., 2024). Whereas nanozymes have better long-term storage, are cost-efficient, and possess higher stability. Therefore, they are increasingly being adopted as a modern alternative, enabling biosensors to achieve improved LOD more quickly while maintaining catalytic efficiency (Altalbawy et al., 2026; Arshad et al., 2022).

## 6 Molecularly Imprinted Polymers (MIPs)

MIPs are synthetic biomimetic receptors engineered to resemble natural molecular recognition. They are fabricated by polymerising functional monomers around a template molecule. After the template is removed, complementary binding cavities remain (Naresh & Lee, 2021). Ren et al. (Ren et al., 2024) developed a fluorescence biosensor using a bacteria-imprinted MIP fabricated with L. *monocytogenes* as the template. The sensor achieved an LOD of 72 CFU/mL and showed high selectivity against several non-target bacteria. The authors highlighted the MIP's low cost, good storage stability, and strong resistance to background fluorescence. In regard to *Salmonella,* Wang, He, et al. (2024) constructed an electrochemical MIP sensor by electropolymerising dopamine around *S. typhimurium* on a screen-printed electrode. The system reached an LOD of 10^1^ CFU/mL and demonstrated excellent selectivity.

MIPs offer high thermal and chemical stability under adverse environmental conditions such as fluctuating pH, temperature, and solvent conditions. This stability arises from their densely cross-linked polymer matrix and strong interactions between functional monomers and target molecules. As a result, MIPs are particularly suitable for challenging environments such as food matrices, unlike protein-based bioreceptors (e.g., antibodies and enzymes) that rely on conformational stability (Kapase et al., 2025). Moreover, they offer low production cost and compatibility with complex samples containing inhibitors or denaturing agents (Kim, Ryu, & Shin, 2025; Kim, Cha, & Kim, 2025).

Despite the successes observed in studies such as Ren et al. (2024) and Wang, He, et al. (2024), MIP-based sensing for foodborne pathogens faces significant challenges. While imprinting of small molecules is well established, whole-cell imprinting remains technically demanding due to the large size and structural complexity of bacteria such as *Listeria* and *Salmonella* (Carvalho et al., 2014; Oludairo et al., 2022; Piletsky et al., 2020). Key challenges include incomplete template removal due to the highly cross-linked matrix, which can lead to template leakage and reduced sensitivity (Kim, Cha, & Kim, 2025;Lamaoui et al., 2024 ; Refaat et al., 2019). Additionally, whole-cell imprinting often results in slow mass transfer kinetics, further limiting sensor performance (Lamaoui et al., 2024; Refaat et al., 2019). The inherent heterogeneity of bacterial cell surfaces may also promote non-specific binding, reducing selectivity and sensitivity in complex food matrices (Lamaoui et al., 2024; Refaat et al., 2019). Furthermore, immobilisation of MIPs onto electrodes remains a challenge and can affect reproducibility (Kim, Ryu, & Shin, 2025). MIPs also have limited intrinsic conductivity, though this can be addressed using conductive nanomaterials such as graphene or multi-walled carbon nanotubes (MWCNTs) to enhance electron transfer and sensor performance (Kim, Ryu, & Shin, 2025).

## 7 Peptides

Peptide bioreceptors consist of short amino acid sequences that may be natural (e.g., antimicrobial peptides) or engineered (e.g., mimetic peptides, peptide aptamers). They occupy a middle ground between biological and synthetic bioreceptors (Naresh & Lee, 2021).

Natural antimicrobial peptides (AMPs) recognise bacterial membranes and have great selectivity toward target pathogen, although lower specificity is exhibited than monoclonal antibodies (Escobar et al., 2023). Additionally, AMPs are susceptible to degradation by food-derived proteases, thus reducing biosensing performance in complex food matrices (Escobar et al., 2023). Compared to engineered peptide, AMPs have been explored less in recent year in the field of L. *monocytogens* and *Salmonella* detection. For example, AMP magainin I was used on micro-capacitive electrodes for detecting several pathogens, including *E. coli*, *Salmonella*, and *Listeria* (Mannoor et al., 2010). While the approach enabled broad, Gram-selective detection at around 10^3^ CFU/mL, it did not lead to widespread follow-up studies, and recent biosensor development has largely favoured synthetic, engineered peptides rather than traditional AMPs.

Engineered peptides on the other hand, are selected through methods such as phage display or AI-guided design display that improve specificity and binding affinity (Kim, Ryu, & Shin, 2025). Their smaller size enhances epitope accessibility, while their stability, lower batch to batch variations, and cost-effectiveness make them attractive alternatives to other bioreceptors such as antibodies (Deshpande et al., 2024; Kim, Ryu, & Shin, 2025; Qin et al., 2025). However, limitations include difficulties in phage display process when using whole bacterial cells due to the intricate bacterial structure (Escobar et al., 2023).

For L. *monocytogenes* detection, Eissa and Zourob (Eissa & Zourob, 2020) developed a multiplexed electrochemical assay based on two custom synthetic peptides that act as substrates for species-specific proteases. The system achieved a low-level detection (9 CFU/mL) and successfully distinguished mixtures containing multiple foodborne bacteria, demonstrating the usefulness of engineered peptides in rapid, low-cost sensing formats. For *Salmonella*, Wang, Ma, et al. (2025) constructed an engineered odorant-binding-protein-derived peptide (OBPP) that served as the bioreceptor to detect isoamyl alcohol, a *Salmonella*-specific metabolic marker. Integrated into an electrochemical sensor, an ultra-low detection limit of 1 ppt was achieved and high selectivity was observed against other volatiles commonly emitted by *Salmonella* or by food matrices.

## 8 Nucleic acid probes & DNA aptamers

Nucleic acid probes are single-stranded DNA or RNA molecules that hybridise with complementary target sequences via Watson–Crick base pairing. This enables high sequence specificity and the production of a measurable biosensor signal (Ferone et al., 2020; Naresh & Lee, 2021). Yang, Wu, et al. (2023) developed a genosensor using DNA probes for the detection of *Listeria monocytogenes*. A highly sensitive LOD of 0.04 nM was achieved, along with high selectivity against closely related *Listeria* species.

Aptamers, while also classified as nucleic acid bioreceptors, they differ from traditional nucleic acid probes. DNA aptamers are short single-stranded DNA oligonucleotides generated via SELEX (Naresh & Lee, 2021). Their ability to fold into 3D structures enables binding to a wide range of targets beyond nucleic acids, including cells, proteins, and small molecules (Centane & Nyokong, 2022; Sezgintürk, 2020). The small size of aptamers (∼1–2 nm), compared to antibodies (∼10–15 nm), provides a vital advantage by enabling higher surface packing density on transducers. This increases the number of available binding sites per unit area, thereby enhancing biosensor sensitivity (Morales & Halpern, 2018). Cao et al. (Cao et al., 2025) developed a CNT-FET biosensor using a mixture of multiple aptamers targeting different epitopes on the surface of L. *monocytogenes*. This strategy provided single-cell detection (∼1 CFU) and strong selectivity against interfering species. These results also illustrate how combining several aptamers can enhance capture efficiency and improve sensitivity compared to single-aptamer systems. For *Salmonella*, Panphut et al. (Panphut et al., 2026) used an aptamer-functionalised magnetic bead system integrated into a superhydrophobic magneto-flow electrochemical sensor. The aptamer enabled selective binding to *S. typhimurium*, achieving a LOD of ∼10 CFU/mL and clear discrimination from non-target bacteria.

Advantages of aptamers include ease of modification against a wide range of targets, high specificity, chemically robust, high affinity, inexpensive and excellent modifiability for controlled immobilisation (Centane & Nyokong, 2022; Yoo et al., 2020). However, limitations include the fact that SELEX is labour-intensive, time-consuming and may have disappointing success rates (Yoo et al., 2020). Additionally, aptamers struggle to distinguish very closely related bacterial strains and they remain susceptible to degradation in real food matrices (Y. Yang et al., 2024). Aptamer binding and structural configuration are also affected by variations in pH and ionic strength. For example, high salt concentrations (e.g., NaCl) can weaken aptamer–target interactions due to electrostatic shielding of the negatively charged DNA backbone (Hianik et al., 2007). Although their small size enables high surface density, it can also make immobilisation challenging on certain substrates, such as nitrocellulose membranes used in test strips for laminar flow assays (X. Pan et al., 2023).

## 9 Bacteriophages

Bacteriophages represent a class of biorecognition elements that naturally recognise and bind to their bacterial hosts (Altalbawy et al., 2026). They specifically infect bacterial cells, and their negatively charged capsids (heads) and positively charged tail fibres allow immobilisation onto sensor surfaces through ionic or electrostatic interactions (Khaleque et al., 2024). Their tail proteins such as receptor binding proteins (RBPs) that are positioned at the ends of the tail provide exceptional molecular specificity, enabling highly selective detection of target pathogens (Richter et al., 2016). The capsids comprise of long amino acid chains. These chains have amine groups at the N-terminus and carboxyl groups at the C-terminus, both groups can act as key targets for immobilising the phage to a biosensor surface (O'Connell et al., 2021). Research suggests ordering the phage layer in a manner of allowing the phages to be perpendicular to the sensing surface with upward positioned tails significantly increases sensing sensitivity, as the RBPs are fully positioned to detect target bacteria (Richter et al., 2016).

Zhang, Zhou, et al. (2025) developed an integrated three-signal (bioluminescent, photothermal, and colorimetric) biosensor using a bacteriophage for the simultaneous detection of live and dead L. *monocytogenes*, achieving a LOD of 1 CFU/mL and 5 CFU/mL, respectively. The biosensor demonstrates high specificity and stability, effectively differentiating between live and dead bacteria. For *Salmonella*, Ding et al. (Y. Ding et al., 2024) employed a phage-derived receptor binding protein (RBP41) from phage T102 as the bioreceptor in an electrochemical DPV sensor. Although a whole bacteriophage was not used, the phage receptor enabled highly specific binding to several *Salmonella* serotypes and achieved a very low LOD of ∼2 CFU/mL, while showing negligible response to non-target bacteria. This example also highlights the growing use of phage proteins rather than whole phages as highly stable and selective bioreceptors.

Advantages include the ability to differentiate live from dead bacteria, high stability across a range of pH and temperatures, low production cost via host replication, and recognition of whole bacterial cells rather than isolated antigens (Escobar et al., 2023). Whereas limitations mainly relate to immobilisation challenges, since the activity of whole bacteriophage is strongly dependent on their orientation, surface conditions, and the immobilisation strategy used (Escobar et al., 2023). Additional drawbacks include practical storage difficulties because phages require bacterial hosts for replication and the possibility of rapid host lysis can reduce the effective signal output (X. Pan et al., 2023).

While phage-based systems have been developed at a research level, currently there are no commercialised phage-based biosensors for the detection of L. *monocytogenes* and *Salmonella*, this is due to limited performance improvements over current techniques (Escobar et al., 2023; Zolti et al., 2022). Nevertheless, the use of bacteriophages as bioreceptors is gaining attraction in the field of biosensing for these foodborne pathogens.

## 10 Whole-cell bioreceptors

Whole-cell bioreceptors employ natural or genetically engineered cells to recognise analytes (Khaleque et al., 2024). These engineered strains often involve membrane modifications, specific antibody integrations, or the use of promoters that activate reporter genes exclusively in the presence of the target analyte, generating measurable outputs (Khaleque et al., 2024; Mendoza et al., 2024). As a result, these bioreceptors exhibit excellent sensitivity and rapid detection capabilities due to their biological amplification mechanisms (Mendoza et al., 2024). Additionally, they are suitable for on-site use, enhancing their practicality in various applications (Ali et al., 2020). However, regulatory restrictions associated with genetically modified organisms (GMOs) pose challenges (A. Singh & Kumar, 2021). Though SimCells (chromosome-free simplified cells) have been proposed to reduce risks of replication and gene transfer, their application to L. *monocytogenes* and *Salmonella* remains unexplored (Rampley et al., 2017). Whole-cell sensors are highly sensitive to environmental factors such as pH, temperature, nutrient availability, and stress, which can compromise their reproducibility (A. Singh & Kumar, 2021). Moreover, complex food matrices can inhibit cell activity, resulting in convoluted responses and diminished selectivity (Hasan et al., 2014).

Currently, only a limited number of studies use whole cells directly as bioreceptors for foodborne pathogen detection. For L. *monocytogenes*, Hadjilouka et al. (Hadjilouka et al., 2020) employed membrane-engineered Vero cells, into an anti-p60 monoclonal antibody via electroinsertion. These modified cells functioned as the bioreceptor within a Bioelectric Recognition Assay (BERA) platform, enabling rapid electrochemical detection after enrichment and achieving an LOD of ∼0.6 log CFU/mL. The system also showed good discrimination from non-target bacteria. For *Salmonella*, Konstantinou et al. (Konstantinou et al., 2023) used a similar approach, engineering Vero cells with monoclonal anti-*Salmonella* antibodies and integrating them into a BERA-based detection system. Following enrichment, the biosensor reached an LOD of ∼1 log CFU/g and distinguished positive from negative meat samples with high accuracy. In both cases, the whole engineered cell rather than the antibody itself acts as the functional bioreceptor, producing a measurable membrane-potential change upon interaction with the target pathogen.

## 11 Transducer introduction

While the bioreceptor determines the specificity of the analyte–binding event, the transducer is responsible for converting this biochemical interaction into a measurable physicochemical signal (Vinay Kumar et al., 2022). This conversion step is fundamental to the performance of the biosensor, influencing analytical characteristics such as sensitivity, detection limit, response time, and suitability for real-world food testing (Sezgintürk, 2020). As a result, the transducer is considered just as crucial as the biorecognition element.

Transducers are generally classified based on the physical principle used for signal conversion (L. Shen et al., 2026). Among the different types developed so far, electrochemical, optical, and piezoelectric (acoustic) transducers remain the most widely applied for detecting foodborne pathogens such as L. *monocytogenes* and *Salmonella* (Awang et al., 2021; Soni et al., 2018). Each type operates via a distinct mechanism and offers unique advantages in terms of miniaturisation, sensitivity, cost, and integration into portable systems (Sezgintürk, 2020).

Increasingly, nanomaterials have become central to improving transducer performance (Muniandy et al., 2019; Wang, Song, et al., 2025). For example, incorporating graphene, carbon nanotubes (CNTs), metal nanoparticles, MXenes, or nanocomposites into electrodes can significantly enhance electron transfer, increase the active surface area, improve bioreceptor loading, and ultimately amplify the resulting signal (Awang et al., 2021; Engku Abd Rahman et al., 2026; Jiang et al., 2025; Muniandy et al., 2019). These material-engineering innovations have driven major improvements in biosensor sensitivity, stability, and rapidity, making them increasingly attractive for food-safety applications (Awang et al., 2021; Wang, Song, et al., 2025).

## 12 Electrochemical transducers

Electrochemical biosensors are among the most widely used contemporary biosensing approaches (H. Wang et al., 2018). These transducers measure electrical changes, typically variations in current, potential, or impedance, that arise from biochemical interactions occurring at the electrode surface (A. Singh et al., 2021). When this surface is modified with nanomaterials such as graphene, gold nanoparticles (AuNPs) and CNTs, the electron-transfer kinetics and local charge distribution are altered, allowing even subtle binding events to generate a detectable signal (Muniandy et al., 2019; A. Singh et al., 2021; Wang, Song, et al., 2025).

**Fig. 3:**
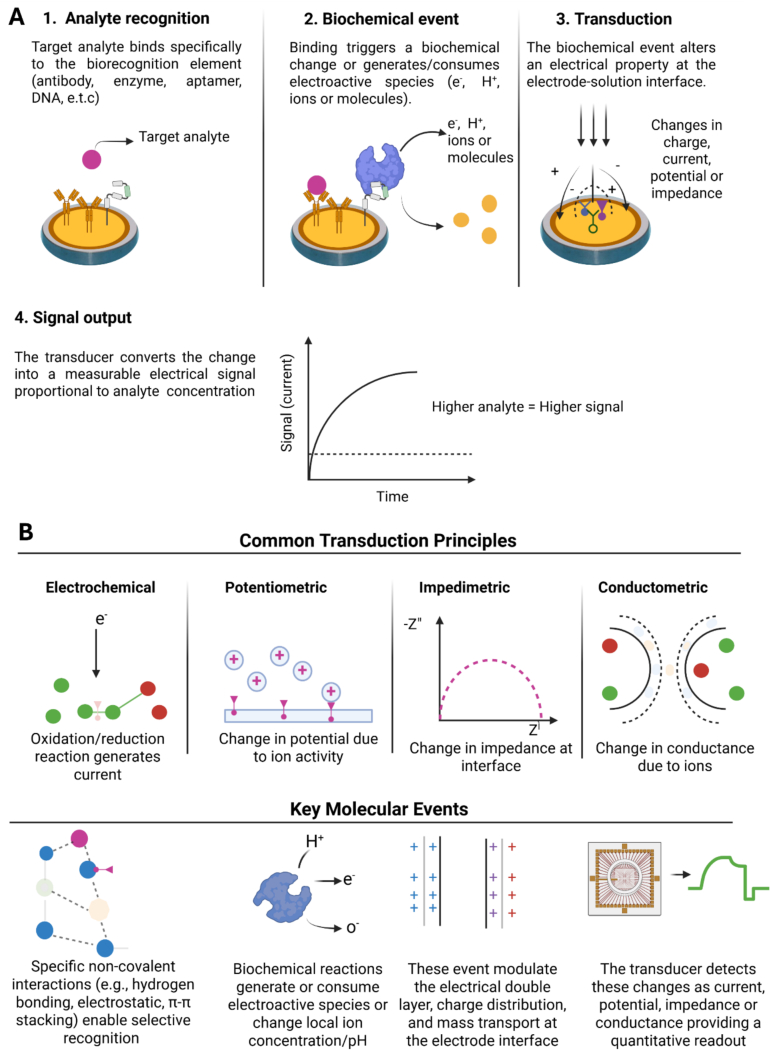
General working principles of electrochemical biosensors and common signal transduction mechanisms. (A) Schematic overview of the electrochemical biosensing process, including analyte recognition, biochemical interaction at the electrode interface, signal transduction, and measurable electrical output. (B) Major electrochemical transduction modes used in biosensors, including electrochemical, potentiometric, impedimetric, and conductometric sensing mechanisms and their associated molecular events. Created with BioRender.com.

In a general electrochemical biosensing process, the target analyte selectively binds to the biorecognition element such as antibody, enzyme or aptamer that is immobilised on the electrode surface (Fig. 3A). This recognition event causes biochemical changes to occur at the electrode–electrolyte interface through the generation or consumption of electroactive species such as electrons, protons, ions, or other molecules. This leads to an alteration in local electrical properties such as the charge distribution, current, potential, or impedance. The transducer then converts these interfacial changes into a measurable electrical signal proportional to analyte concentration, where higher analyte levels produce stronger sensor responses (Sezgintürk, 2020).

Several electrochemical transduction mechanisms may be utilised depending on the sensing strategy (Fig. 3B). Amperometric and voltametric sensors detect current generated through oxidation–reduction reactions of electroactive species, whereas potentiometric sensors monitor changes in interfacial potential caused by ion activity. Impedimetric sensors measure variations in interfacial impedance or charge-transfer resistance at the electrode surface, while conductometric sensors detect changes in conductivity resulting from ionic transport within the sensing environment (Sezgintürk, 2020). At the molecular level, these sensing approaches rely on selective non-covalent interactions and biochemical reactions that modulate electron transfer, local pH, ion concentration, or the electrical double layer at the electrode interface, ultimately producing measurable outputs such as current, voltage, impedance, or conductance (Grieshaber et al., 2008).

Electrochemical biosensors are especially suitable for foodborne pathogen detection (Khaleque et al., 2024) because they rely on inexpensive electrode materials (e.g., carbon-based substrates), require minimal instrumentation, allow for highly portable designs such as lab-on-chip formats, and often enable rapid, label-free detection (Banicod et al., 2025; Engku Abd Rahman et al., 2026; Muniandy et al., 2019). These characteristics make them promising candidates for on-site monitoring in food-production settings.

However, their performance can be affected by real-sample conditions (Engku Abd Rahman et al., 2026). Non-specific adsorption, fouling, and matrix effects from complex food samples may reduce reproducibility (Banicod et al., 2025; Muniandy et al., 2019; A. Singh et al., 2021). Despite these limitations, continued advances in surface chemistry, antifouling coatings, and nanomaterial engineering are steadily improving electrochemical biosensor reliability and robustness (A. Singh et al., 2021). An overview of the typical examples of electrochemical biosensors is depicted in Table 2.

**Table 2:** Typical examples of electrochemical biosensors for *Listeria* and *Salmonella.*

Pathogen	Mode / Electrode	LOD	Matrix	Ad (+)	Dis (−)	References
*L. monocytogenes*	SPCE/Ti₃C₂Tₓ MXene–CuNP, aptamers	3 CFU/mL	Milk	High sensitivity and stability, dual pathogen detection, strong signal amplification	Multi-step fabrication, MOF aptamer gating system can be difficult to scale	(Jiang et al., 2025)
*L. monocytogenes*	ECL, Pt/ZnO–3DNGH, aptamers	5 CFU/mL	Pork, milk	Tested in two food matrices, ECL provided high sensitivity and low background	Complex build difficult to scale, Pt use as an electrode is expensive	(Chen et al., 2022)
*L. monocytogenes*	EIS, q-CNTs, P100 phage	10 CFU/mL	Chicken broth	High sensitivity, 8-min assay, simple low cost and portable platform, label free EIS detection	Poor long-term stability (∼2 weeks), higher LOD	(Zolti et al., 2024)
*S. typhimurium*	Voltammetry, rGO-CNT/GCE, aptamer	10 CFU/mL	Chicken	Fast (10 min), selective	Aptamer sensitive to environment	(Appaturi et al., 2020)
*S. typhimurium*	Paper sensor, CNC	3.5 CFU/mL	Eggs, chicken, pork	Very sensitive, large surface area	Ag/AgCl ink unstable	(Jampasa et al., 2025)

## 13 Electrochemical biosensors for L. *monocytogenes*

Jiang et al. (Jiang et al., 2025) produced an electrochemical biosensor for the simultaneous detection of two foodborne pathogens, *L. monocytogenes* and *Staphylococcus aureus* (*S. aureus*). Screen-printed carbon electrodes (SPCE) were modified with Ti_3_C_2_T_x_ MXene nanoribbons/copper nanoparticles (Ti_3_C_2_T_x_ NR/Cu) through a self-reduction process, in which Cu^2+^ ions are reduced from CuCl_2_ to Cu nanoparticles (CuNPs). Ti_3_C_2_T_x_ MXene possesses unique properties, for instance, being innately hydrophilic, having a particularly high surface area, and exhibiting excellent electrical properties. They also possess special non-bonding Ti states that allow specific electron transfer and direct reduction of metal ions, hence the self-reduction process allowing the formation of CuNPs in situ on the MXene surface. This property is important for signal amplification, as it enables the formation of the Ti_3_C_2_T_x_ NR/Cu nanohybrid. The Ti_3_C_2_T_x_ xNR also possesses a large active site, thus leading to the capability of loading further CuNPs. Moreover, the addition of CuNPs was conducive to increasing the conductivity of Ti_3_C_2_T_x_ NR, thereupon further enhancing signal amplification.

Within this study, aptamers specific to each bacterium were attached as biological surface gatekeepers on UiO-66. UiO-66 is a zirconium-based metal-organic framework (MOF) made of Zr_6_O_4_(OH)_4_ clusters connected by 1,4-benzenedicarboxylate (BDC) ligands (Lee et al., 2021). The use of UiO-66 in this study is for the purpose of confining probe molecules such as ferrocene (Fc) for the detection of L. *monocytogenes* and methylene blue (MB) for the detection of *S. aureus* within the nanopores. This nanoscale material encompasses the role of a porous host framework with high specific surface area and porosity, whilst also providing a stable porous structure; this makes it suitable for trapping the probe tags without affecting the MOF structure.

As Fc is confined within the nanopores of the UiO-66 MOF, when a bacterium such as L. *monocytogenes* is absent, there is minimal differential pulse voltammetry (DPV) signal produced. However, when L. *monocytogenes* is present, the bacterium binds to the target aptamers, opening the biological gatekeepers and releasing the encapsulated probe tags, which are then measured at the modified electrode. Thereupon, resulting in higher DPV signals, with higher bacterial concentration corresponding to higher signal intensities. With the possibility of different signal peaks representing different bacterial binding, this allows detection of multiple bacteria within the same biosensor. As mentioned previously, the addition of CuNPs as nanoparticles to the Ti_3_C_2_T_x_ NR/SPCE enhanced these electrochemical signals, henceforth significantly improving the biosensing action.

The detection limit achieved within this study was 3 CFU/mL for L. *monocytogenes* and 2 CFU/mL for *S. aureus*. The real-life sample tested in this study was milk, and the incubation time used for the biosensing assay was 30 min, with a linear range spanning from 10^1^ to 10^8^ CFU/mL. The biosensor also exhibited significant current signal changes for *S. aureus* and L. *monocytogenes*, while showing minimal responses to other tested pathogens, including *Shigella flexneri*, *Salmonella enteritidis*, *Escherichia coli*, and *Vibrio parahaemolyticus*. Tests in spiked milk yielded recovery rates of 91.3–97.6%, demonstrating practical reliability. The device maintained over 91% of its initial current after 56 days, indicating strong long-term stability. This biosensor demonstrates a strong potential of Ti_3_C_2_T_x_ MXene nanoribbons/copper nanoparticles nanomaterials for the sensitive dual detection of foodborne pathogens, offering a strong promise for advancing the food industry.

In another work, Chen et al. (Chen et al., 2022) built an electrochemiluminescence (ECL) biosensor composed of platinum (Pt) electrodes modified with a zinc oxide three-dimensional nitrogen-doped graphene composite (ZnO-3DNGH) for the detection of *L. monocytogenes*. Graphene oxide (GO) is used as the initial material for the construction of the biosensing interface, as it offers substantial benefits suited for a biosensor, such as increased chemical stability and surface area. However, the researchers in this study took a further step by converting this flat two-dimensional (2D) GO into a three-dimensional porous graphene-derived structure. This transformation incorporated further improvements to the existing properties of GO, making it more suitable for optimisation of the biosensor interface (Hu et al., 2024). For example, this transformation prevents graphene sheets from agglomerating and restacking due to strong van der Waals interactions, as seen in 2D GO (Bagoole et al., 2017). Thus, this transformation expands the surface area even further for the luminophore Ru(bpy)3^2^+ and increases the loading capacity for high-density materials such as aptamers, thereby improving L. *monocytogenes* recognition at the biosensing surface (Bagoole et al., 2017).

Subsequently, the graphene-derived 3D hydrogel is subjected to nitrogen doping, where heteroatoms are incorporated into the graphene lattice to modify its electronic structure. This modification enhances the intrinsic electrical conductivity of the electrochemiluminescence (ECL) sensing platform (Joucken et al., 2015; Talukder et al., 2021). Finally, ZnO nanoparticles, serving as the catalytic component, are integrated with the 3DNGH to form a ZnO–3DNGH nanocomposite, which is subsequently deposited onto the Pt electrode. The incorporation of this metal oxide enhances electron transfer and structural stability, thereby improving the overall electrochemical performance of the biosensor.

For luminescent signal generation, TPA (tripropylamine) acts as the co-reactant for Ru(bpy)3^2^+. Thus, when electrochemical oxidation occurs at the electrode, excited ruthenium species emit light. Meanwhile, an amino-modified aptamer is used as the biorecognition element and immobilised onto the ZnO-3DNGH/Ru(bpy)3^2^+ surface. When L. *monocytogenes* binds to the aptamer, it results in an increase in steric hindrance. This in turn modifies the interfacial properties of the electrode surface. As the ECL Ru(bpy)3^2^+/TPA mechanism is dependent on effective co-reactant oxidation and electron transfer at the electrode surface, the binding of L. *monocytogenes* inhibits this process due to surface obstruction (Valenti et al., 2016). Henceforth, a signal-off system is displayed, where greater bacterial binding leads to a decrease in ECL intensity.

This biosensor achieved an LOD of 5 CFU/mL, generating results within 30 min over a linear range of 15–1.5 × 10^7^ CFU/mL. Real-sample testing was performed in pork and milk products, producing recovery rates of 91.4–104.2% and 96.7%–103.6%, respectively. The selectivity test was performed against *Bacillus subtilis*, *Shigella flexneri*, *S. aureus*, *Salmonella* and *E. coli*. Minimal change was observed in the signal when tested with these other bacteria, with only significant changes shown when L. *monocytogenes* was present. The study highlights the potential of ZnO-3DNGH as a nanocomposite for the use of electrochemical biosensing of L. *monocytogenes*.

A different strategy was employed in a study by Zolti et al. (Zolti et al., 2024). *L. monocytogenes* was detected using electrochemical impedance spectroscopy (EIS) with commercially available screen-printed electrodes (SPE) modified with quaternized carbon nanotubes (q-CNT). The prior steps before the modification of the SPE required the transformation of carboxylic acid functionalised multiwalled carbon nanotubes (COOH-CNT) into q-CNT. Although COOH-functionalized carbon nanotubes (COOH-CNTs) exhibit excellent properties, including high mechanical strength and thermal and electrical conductivity, quaternization was required in this study to facilitate functionalization with the bioreceptor, the P100 bacteriophage (Wang, Wang, et al., 2025). COOH-CNT have a negative charge; however, when quaternized, ammonium groups are introduced onto the nanotube surface, thereby creating a permanent positive charge (Huang et al., 2013; Wang, Wang, et al., 2025). This positive charge is critical, as the bacteriophage has a negatively charged head and a positively charged tail (Maciel et al., 2023). Henceforth, the positively charged q-CNT attracts the head of the P100 phage, orienting it onto the biosensing interface via electrostatic forces of attraction, whilst the tail remains available to capture L. *monocytogenes* (H. Yang et al., 2025).

Meanwhile, 1-pyrenebutyric acid succinimidyl ester (PBSE) in this study was used as a crosslinker and tethering agent to allow attachment of the phage to the q-CNT-modified SPE. The pyrene groups in PBSE allow for strong non-covalent anchoring through π–π interactions with the carbon nanotube surface (Lei & Ju, 2010), whilst the succinimidyl ester groups react with amine groups on the P100 phage surface (Lei & Ju, 2010; O'Connell et al., 2021). Therefore, the q-CNT surface aligns the bacteriophage through electrostatic interactions, while PBSE provides a stable chemical tether to hold the phage on the SPE surface.

Once L. *monocytogenes* binds to the P100 phage, the interfacial charge-transfer properties of the electrode changes, allowing EIS measurements to detect this binding. The EIS system employed in this work measures electron transfer between the modified SPE and the redox couple [Fe(CN)_6_]^4^−/[Fe(CN)_6_]^3^−. When *L. monocytogenes* binds to the phage, the whole bacterial cell impedes the interfacial layer, causing more hindered electron exchange of the redox probe with the q-CNT/SPE surface. This results in an increase in the charge-transfer resistance (RCT). As the concentration of the bacteria increases, the value of RCT also increases.

The biosensor achieved an LOD of 10 CFU/mL in 1% chicken broth with a fast 8-min incubation. Recovery rates in real samples ranged from 87 to 96%. A portable version was successfully integrated into a flow-cell system using SPEs, indicating good potential for production-line monitoring. Specificity tests against *E. coli* and *S. typhimurium* displayed high specificity only to L. *monocytogenes*. The biosensor showed stability for up to two weeks, after which the signal declined. Also, a no-phage test was conducted, showing that without the bioreceptor, the RCT values remained nearly constant with no significant change even as L. *monocytogenes* concentration increased. This further emphasised the specificity of the bacteriophage used. Overall, the q-CNT/SPE EIS-based biosensor utilised in this research highlights strong advancement in detecting L. *monocytogenes* based on a sensitive, inexpensive and portable system.

## 14 Electrochemical biosensors for *Salmonella*

For detecting *Salmonella*, specifically *S. typhimurium,* a study conducted by Appaturi et al. (Appaturi et al., 2020) developed an ssDNA/rGO-CNT/GCE aptasensor. This biosensor uses a specific nanomaterial composed of reduced graphene oxide (rGO) and carbon nanotubes (CNT) were placed onto a glassy carbon electrode (GCE). This incorporation was chosen because rGO has a higher current density and is not easily degraded; however, it has limitations in terms of its structure, which is composed of sheets that can be easily restacked due to van der Waals forces, thereby decreasing the surface area. Thus, a spacer, here represented by the CNT, is required to prevent re-stacking and consequently increase the surface area for binding of the bioreceptor (ssDNA aptamer). This design enabled the aptasensor to detect as low as 10 CFU/mL, demonstrating good sensitivity. In addition to that it also enabled rapid detection, providing results within 10 min when tested in raw chicken meat samples. The strong selectivity of the aptasensor was demonstrated when tested against other *Salmonella* serovars, which showed signals similar to *S. typhimurium*. In contrast, it has high specificity, which was confirmed by the much weaker signals produced when tested against non-*Salmonella* species. Despite the promising results achieved in this study, it demonstrated some limitations regarding the aptamer design, as its activity can be easily affected by environmental factors such as temperature, pH, etc., which in turn affect the overall performance of the aptasensor.

Unlike the earlier biosensor design, the paper-based biosensors with cellulose nanocrystals (CNC) performed better in terms of its sensitivity and specificity (Jampasa et al., 2025). This is attributed to the CNCs, which exhibit higher conductivity than rGO/CNT due to their high functional groups, particularly COOH and OH, that contribute to increased electron density. Additionally, CNCs double the surface area by increasing the binding cites for bioreceptor on the surface of the biosensor. All of these properties helped the biosensor to achieve a limit of detection of 3.5 CFU/mL and exhibited a high specificity, as no signal was observed compared with the signal from *S. typhimurium* during the testing against other bacteria. Also, it demonstrated fast detection, providing results within 30 min in food samples like eggs, chicken, and pork. However, the study noted that the use of the Ag/AgCl ink to make the electrode pads was not ideal for long-term storage, as the Ag can be oxidized when exposed to air or moisture, resulting in decreased conductivity, thereby impairing the sensor's overall performance.

## 15 Critical analysis

Across the various electrochemical studies mentioned, specific transduction mechanisms are employed, including redox-probe-mediated signal amplification (Jiang et al. (Jiang et al., 2025), electrochemiluminescence (Chen et al., 2022), and impedance-based surface blocking (Zolti et al., 2024), each introducing different interfacial constraints and performance trade-offs.

In Jiang et al.'s. (Jiang et al., 2025) study, the use of Ti₃C₂Tx Maxene nanoribbons have an advantage in not requiring the addition of an independent chemical reducing agent to form CuNPs, as the nanomaterial itself allows for a self-reduction mechanism. This is beneficial not only for increasing the active sites of the Ti₃C₂TxNR material, but the coupling with CuNPs also enhances the conductivity of the material, thus amplifying signal responses and resulting in increased biosensor sensitivity. Additionally, the study showed that the nanoribbon structure, more precisely, provides the increased active sites, while the CuNP coupling enhances conductivity. Therefore, from a mechanistic perspective, the nanoribbon morphology increases the available surface area and exposure of active sites. This leads to improvements in the interaction between the target bacteria and the sensing interface. Compared to Chen et al. (Chen et al., 2022) and Zolti et al. (Zolti et al., 2024), where signal changes are largely governed by interfacial obstruction or ECL disruption, Jiang et al.'s. (Jiang et al., 2025) system relies more heavily on enhanced surface reactivity and electron-transfer pathways to amplify detection signals.

Although SPCEs possess specific benefits such as being cost-effective, having simple production processes, and allowing mass production, Jiang et al.'s. (Jiang et al., 2025) highlighted that plain SPCE biosensors without Ti₃C₂TxNR/Cu modification resulted in insufficient signals and biosensing capabilities. The combination of MXene-based Ti₃C₂TxNR/Cu nanomaterials and the aptamer bio-gate/MOF mechanism with SPCE leads to an overall increase in material capability, detection of multiple foodborne pathogens with higher sensitivity. This demonstrates strong promise for advancing the detection of foodborne pathogens and benefiting the food industry. However, in contrast to Zolti et al. (Zolti et al., 2024) simpler SPE-based system, Jiang et al. (Jiang et al., 2025) multi-component modification introduces increased fabrication complexity.

The use of this modification in Jiang et al. (Jiang et al., 2025) may present challenges for large-scale manufacturing. The in situ self-reduction process for CuNP formation and the multiple surface modification steps needed for electrode preparation can lead to variability and lower reproducibility during mass production. From a practical and interfacial viewpoint, achieving uniform nanomaterial deposition and consistent surface coverage across multiple SPCEs can be difficult, which may impact sensor performance and reliability. Therefore, although Ti₃C₂TxNR/Cu modification improves detection capability, translating it to large-scale production can be more challenging.

Compared to other electrochemical studies mentioned, in Jiang et al.'s. (Jiang et al., 2025) study, the use of a multiplexed aptasensor provides a key advantage in the ease of replacement of aptamers for the simultaneous detection of different bacteria within the same biosensing platform. This enables wide applicability for advancing food safety, as more than one type of bacteria can be present within the real-life food sample (Nyenje et al., 2012). Furthermore, the use of aptamers as biological molecular gatekeepers, combined with highly porous MOF materials such as UiO-66, results in a highly stable and sensitive system. The porous MOF structure contributes physically by increasing loading capacity and facilitating mass transport of probe molecules within the sensing layer, thereby enhancing interaction efficiency at the electrode interface. In comparison, Chen et al. (Chen et al., 2022) 3D graphene structure similarly improves surface area and interaction efficiency, whereas Zolti et al. (Zolti et al., 2024) relies less on internal porosity and more on surface blocking effects for signal generation. Hence, the analytical benefit of multiplex detection is strengthened by the physical properties of the MOF structure, which enhance sensor performance at the interfacial level.

The Ti₃C₂TxNR/Cu biosensor produced in Jiang et al. (Jiang et al., 2025) study showed that hindrance from co-existing substances, for instance Ca^2+^, K^+^, Mg^2+^, vitamin A, vitamin D, triglycerides, and riboflavin within real food samples, did not impact the detection of L. *monocytogenes* or *S. aureus*. This is a very critical advantage because, in real-life samples, complex food matrices with a high potential for bio-interference can cause signal interference when detecting bacteria. Milk, for instance, is a challenging matrix for electrochemical biosensing due to electrode fouling and biofouling, as well as reduced selectivity and specificity (Y. Pan et al., 2024; Sangubotla et al., 2026). Within milk, whey proteins, fat globules, and casein micelles can lead to biofouling challenges because the adsorption of these substances can form an insulating layer, which restricts electron transfer kinetics whilst also altering electrode interfacial properties. This is specifically challenging in voltametric methods, such as DPV, as access of electroactive species to the electrode surface drastically impacts peak potential and current (Sangubotla et al., 2026). This fouling represents a physical barrier that can limit diffusion and access of redox species to the electrode surface. Therefore, it is important for electrochemical biosensors to incorporate antifouling properties to minimise the impact of complex food matrices on the electrode surface and maintain accurate detection. While Jiang et al. (Jiang et al., 2025) demonstrate strong resistance to such interference, Chen et al. (Chen et al., 2022) highlights similar fouling challenges in meat matrices, and Zolti et al. (Zolti et al., 2024) address this issue differently through surface blocking detection and matrix-dependent signal enhancement.

In general, in a TPA-based ECL mechanism, heterogeneous electron-transfer kinetics and the electrode surface are vital, as they impact factors such as co-reactant oxidation, ECL efficiency, signal-to-noise ratio and stability (Valenti et al., 2016). Therefore, in Chen et al.'s. (Chen et al., 2022) study, the ZnO-3DNGH-modified Pt electrode nanocomposite represents a design attempt to improve the electrode surface for efficient electron transfer, loading of Ru(bpy)₃^2+^, aptamer immobilisation and signal generation, leading to improved L. *monocytogenes* biosensing detection. From an interfacial perspective, this highlights the importance of surface structure and electron-transfer pathways in improving ECL signal outputs. However, Chen et al. (Chen et al., 2022) system of relying on maintaining the efficient interfacial electron transfer for light emission makes it more sensitive to surface obstruction, compared to Jiang et al.'s. (Jiang et al., 2025), where signal amplification is driven by redox probe release.

Chen et al. (Chen et al., 2022) enhanced the biosensing interface by using three-dimensional (3D) graphene structures, which increased the surface area and facilitated interaction between the analyte and the sensing interface. The 3D structure improves mass transport and provides more accessible binding sites, enhancing interaction efficiency at the electrode interface. However, utilising Pt as the base electrode substrate can be considered a possible limitation under aqueous Ru(bpy)₃^2+^/TPA ECL environments. This is due to the surface oxidation that Pt is susceptible to at high anodic potentials, which can lower ECL efficiency and alter interfacial electron transfer (Valenti et al., 2016). Therefore, although the Pt electrode offers a conductive base, it might not always be the most ideal substrate for amplifying Ru(bpy)₃^2+^/TPA ECL output in water. Additionally, the binding of bacteria causes steric hindrance at the electrode surface; this can physically obstruct co-reactant and electrode interactions, further reducing signal intensity (Martínez-Periñán et al., 2020). This may also partly contribute to the higher LOD reported in this study (5 CFU/mL) compared with other electrochemical studies with lower LODs, for example, in Jiang et al.'s. (Jiang et al., 2025) study (3 CFU/mL), although such comparisons should be interpreted cautiously due to differences in sensing mechanisms and material design. In contrast, Zolti et al. (Zolti et al., 2024) system is less dependent on electron transfer efficiency and instead relies on impedance changes caused by physical surface blocking. This mechanistic dependence on efficient electron-transfer pathways also introduces a limitation not observed in EIS-based systems such as Zolti et al. (Zolti et al., 2024), because any surface oxidation or fouling on Pt electrodes can impact ECL intensity, whereas EIS-based biosensing remains sensitive even when the electrode surface is not fully accessible.

A major advantage of Chen et al.'s. (Chen et al., 2022) study is that biosensor testing occurred in both milk and pork products. In contrast to milk, meat such as chicken and pork presents a more heterogeneous and denser environment, as it contains complex structural proteins such as collagen, actin and myosin, as well as intramuscular fats (Doerscher et al., 2004; Malgwi et al., 2022; Pérez-Juan et al., 2008) . Furthermore, meat products can contain oxidation by-products and endogenous enzymes, which may interfere with biosensing surfaces (Tsegay et al., 2025). This can lead to electrode biofouling due to non-specific adsorption of proteins and other matrix components onto the electrode surface, thereby obstructing the electrode surface and altering interfacial properties. Such non-specific adsorption can also result in false-negative or less reliable results, whilst impacting signal intensity and stability (Harris et al., 2021; Song et al., 2023; Tsegay et al., 2025). This fouling results in steric hindrance and surface blocking, limiting access of electroactive species to the sensing interface.

When considering the food industry, portability and on-site use are vital (Zhang, Ma, et al., 2025). Compared to other studies mentioned in the electrochemical biosensor examples, only Zolti et al. (Zolti et al., 2024) performed flow experiments, where the SPE was placed inside a microfluidic flow cell and liquids were pumped across the sensing surface using a syringe pump. This is critical for the food industry, as the use of a flow system eliminates limitations such as reliance on manual pipetting and instead introduces advantages such as portability and operational flexibility (Zhang, Ma, et al., 2025). Zolti et al. (Zolti et al., 2024) demonstrated that, under flow conditions, the sensor response varied proportionally with changes in the concentration of *Listeria monocytogenes*. Although a 10% reduction in response was observed each time the flow rate doubled, due to reduced binding time, the EIS signal remained detectable and concentration dependent. This highlights the role of mass transport, where reduced interaction time between L. *monocytogenes* and the sensing surface directly influences signal intensity.

Compared to the other electrochemical biosensing studies mentioned, the study by Zolti et al. (Zolti et al., 2024) is the only one that uses EIS-based impedimetric biosensing. The use of EIS highlights its strength as a sensitive technique for detecting small interfacial changes without requiring the bacteria to be electroactive (Moghtader et al., 2016). Captured L. *monocytogenes* cells form a surface-bound layer that acts as a mechanical and electrical barrier. Due to their large size relative to the redox probes, these layers occupy electroactive surface area, reducing probe access and hindering electron-transfer pathways (Inico et al., 2025; Jamshidi & Zeinali, 2019; Moghtader et al., 2016). Furthermore, the use of commercially available screen-printed electrodes (SPEs) supports industrial application, as they are cost-effective compared with expensive materials such as Pt used in Chen et al. (Chen et al., 2022) or the complex, multi-component nanomaterial systems (Valenti et al., 2016). Therefore, EIS offers advantages such as portability and cost-effectiveness.

To conclude, in both *Listeria* and *Salmonella* electrochemical transducer examples, a clear trend emerges: nanomaterial-enhanced electrodes consistently achieve low detection limits, rapid detection times, and strong selectivity. High-surface-area materials (e.g., MXenes, CNTs, graphene derivatives) and conductive nanocomposites significantly improved the electron transfer and enable dense bioreceptor immobilisation, contributing to excellent sensitivity. However, despite strong laboratory performance, real-world adoption remains limited as seen in the studies above. Challenges include complex multi-step fabrication of electrodes, expensive nanomaterial synthesis despite inexpensive base electrodes and susceptibility to food-matrix interference. Moreover, scale-up difficulties for mass production and stability concerns, particularly for aptamer-based systems also remains as problems regarding electrochemical biosensors. Thus, while electrochemical biosensors show exceptional potential for foodborne pathogen detection, especially for achieving low LOD values, their translation into deployable industrial devices requires more emphasis on robustness, manufacturability, and simplified fabrication.

## 16 Optical transducers

Optical transducers represent another major class of biosensing technologies, relying on the interaction between light and matter to monitor pathogen-related binding events (Sezgintürk, 2020; Soni et al., 2018) . Changes in optical properties such as fluorescence intensity, refractive index, absorbance, or Raman scattering occur when the analyte interacts with the recognition layer, enabling the conversion of a biological event into a measurable optical output (Wang, Jia, & Lin, 2024). A variety of detection mechanisms fall under this category, including surface-enhanced Raman scattering (SERS), surface plasmon resonance (SPR), colourimetric assays, and fluorescence-based systems (Sezgintürk, 2020). Despite the technical diversity, all optical biosensors operate under the shared principle of translating a biochemical recognition event into an optical signal that correlates with the analyte concentration (Wang, Jia, & Lin, 2024).

Optical biosensors offer key advantages, such as high sensitivity, real-time and possible label-free monitoring as well as the ability to produce visually interpretable signals (Sezgintürk, 2020; Soni et al., 2018). Fluorescence and colourimetric platforms are particularly suited for rapid screening and potential field deployment, as the resulting signals may be visible to the naked eye without the need for sophisticated instrumentation (Naresh & Lee, 2021; Wang, Jia, & Lin, 2024). By contrast, methods such as SERS and SPR can achieve exceptional detection limits but typically require stable light sources, precise alignment, and controlled conditions, positioning them more firmly within laboratory settings (Sezgintürk, 2020; Wang, Jia, & Lin, 2024). Limitations involving instrument complexity, alignment requirements, and optical instability can make optical systems more costly or difficult to deploy in compare to electrochemical biosensors (Banicod et al., 2025; Sezgintürk, 2020). Even so, recent innovations have focused on miniaturisation, enhanced signal amplification, and more robust optical materials, increasing their practical relevance to food safety detection (Wang, Jia, & Lin, 2024). The typical optical based biosensing platforms for L. *monocytogenes* and *Salmonella* are summarised in Table 3.

**Table 3:** Typical examples of optical biosensors for *Listeria* and *Salmonella.*

Pathogen	Mode / Optical Platform	LOD	Matrix	Ad (+)	Dis (−)	References
*L. monocytogenes*	Ratiometric fluorescence (FRET + EXPAR), aptamer-based	0.56 CFU/mL	Shrimp, fish, milk	Low LOD, self-calibration, high selectivity	Multi-step prep, requires lab-grade optics	(Wang, Wu, et al., 2025)
*L. monocytogenes*	Label-free photoluminescence, 1D ZnO–Au nanocomposite, antibodies	8.3 × 10^2^ CFU/mL	–	Fast (15 min), simple readout	No real-sample validation, lower sensitivity	(Myndrul et al., 2024)
*L. monocytogenes*	Fluorescent QD (CdSe@ZnS) immunosensor + magnetic beads	0.26 CFU/mL	Milk, fish	Very high capture efficiency, strong QD signal	QD stability concerns, needs further robustness testing	(Thi Le et al., 2025)
*S. typhimurium*	Colorimetric CMCS–aptamer–AuNP composite	16 CFU/mL	Milk	Simple, good specificity, visible color change	Poor resolution across wide range	(Yi et al., 2019)

## 17 Optical biosensors for L. *monocytogenes.*

Recent work on optical detection of L. *monocytogenes* demonstrates how fluorescence- and photoluminescence-based platforms can achieve extremely low detection limits. One example is the ratiometric fluorescence aptasensor developed by Wang, Wu, et al. (2025), which operates via Förster Resonance Energy Transfer (FRET) driven by amplification fragments produced through EXPAR. The dual emission ratiometric design provides self-calibration, increasing the reliability of the optical output and helping to counteract environmental fluctuations that commonly affect fluorescence measurements. The use of *Listeria*-specific aptamers ensured high selectivity, and the platform achieved an exceptionally low LOD of 0.56 CFU/mL with a wide linear range of 1.0 × 10^1^ to 1.0 × 10^6^ CFU/mL. The system also performed effectively in challenging food matrices, including shrimp, fish, and milk. While analytically impressive, the sensor's reliance on multi-step preparation and laboratory-grade optical instrumentation limits its current suitability for field use and raises questions regarding scalability.

In contrast to this more complex, amplification-assisted approach, Myndrul et al. (Myndrul et al., 2024) presented a label-free photoluminescence immunosensor based on a one-dimensional ZnO–Au nanocomposite. Here, the strong photoluminescent properties of ZnO nanostructures provided the primary optical signal, while Au nanoparticles facilitated antibody immobilisation. Binding of L. *monocytogenes* cells induced a reduction in photoluminescence (PL) intensity, yielding a detection limit of 8.3 × 10^2^ CFU/mL with a rapid 15-min response. This study highlights how nanocomposites can enhance PL-based sensing performance, though the platform has not yet been validated in real food samples and was demonstrated only in a controlled flow-cell environment.

A further fluorescence-based strategy was demonstrated by Le et al. (Thi Le et al., 2025), who integrated monoclonal-antibody-coated magnetic beads with core–shell CdSe@ZnS quantum dots (QDs). The magnetic beads, selected to be similar in size to L. *monocytogenes*, enabled highly efficient cell capture, achieving approximately 96% recovery in just 10 min. The strong fluorescence emitted by QDs amplified the optical signal, allowing for an exceptionally low LOD of 0.26 CFU/mL and a linear range spanning 1 to 10,000 CFU/mL. The entire assay was completed within 35 min, and specificity toward L. *monocytogenes* was high, showing negligible cross-reactivity with *E. coli*. Although highly promising, the stability of bioconjugated QDs remains an open question, and further long-term studies are needed to evaluate their real-world robustness.

## 18 Optical biosensors for *Salmonella.*

In the case of *Salmonella* detection, Yi et al. (Yi et al., 2019) developed a carboxymethyl chitosan (CMCS)-Apt-AuNP colorimetric biosensor. This biosensor is composed of CMCS, an aptamer, and gold nanoparticles, which together form a composite that aids the detection of *Salmonella,* specifically *S. typhimurium*. In this design, CMCs enhance detection efficiency by specifically acting as a carrier that transports the aptamer-AuNP conjugates across the *Salmonella* membrane to access its DNA. In terms of sensitivity, the gold nanoparticle plays a significant role by amplifying the optical signal, meaning that the color change upon *Salmonella* detection becomes stronger and more visible; therefore, the overall sensitivity is improved. Because of this design, the biosensor achieved a limit of detection of 16 CFU/mL and demonstrated a good specificity when tested against other bacteria. Although this biosensor provides promising results, it detects *S. typhimurium* over a wide concentration range, meaning that even small changes in the bacterial concentration do not result in large differences in the change of color, suggesting that the biosensor has poor detection resolution.

Overall, the biosensing strategies described highlight the use of optical platforms, nanomaterial integration, and hybrid capture methods to achieve lower detection limits for L. *monocytogenes* and *Salmonella*. For *Listeria*, optical platforms appear particularly well explored and have achieved some of the lowest LOD values reported across all transducer types, often reaching sub-CFU/mL levels. In contrast, optical based *Salmonella* detection, while promising, remains less developed, possibly due to the genetic and antigenic diversity across *Salmonella* serovars, which can complicate bioreceptor design and validation. Despite challenges related to optical alignment, instrumentation complexity, and stability, recent advances suggest that optical biosensors, particularly fluorescence-based and nanomaterial-enhanced systems, are becoming increasingly competitive and may play a central role in next-generation foodborne pathogen monitoring.

## 19 Piezoelectric transducers

The third major type of biosensing system is the piezoelectric (or acoustic) transducer (Soni et al., 2018). These systems operate on the piezoelectric effect, where certain crystalline materials generate an electrical signal when subjected to mechanical stress or vibration (Sezgintürk, 2020). In biosensing, this principle is used to detect the mass or viscoelastic changes that occur when the target pathogen binds to a bioreceptor immobilised on the surface of a vibrating crystal (Naresh & Lee, 2021; Senturk et al., 2018). This binding event alters the resonance frequency of the crystal, and the magnitude of the frequency shift is directly proportional to the mass of the adsorbed analyte (Soni et al., 2018; Suman & Kumar, 2008). Quartz Crystal Microbalance (QCM) technology is the most widely used piezoelectric platform for detecting foodborne pathogens (Suman & Kumar, 2008; H. Wang et al., 2018).

Piezoelectric biosensors are particularly well suited to detecting whole bacterial cells and large biomolecules, as their detection mechanism is based on mass loading rather than electrochemical charge transfer or optical properties (H. Wang et al., 2018). Although many biosensor formats offer label-free detection, piezoelectric sensors are inherently label-free by design and do not require additional electrochemical or fluorescent tags, which simplifies assay preparation (Sezgintürk, 2020; H. Wang et al., 2018). However, they are also sensitive to environmental factors such as viscosity, temperature, and humidity, all of which can influence crystal oscillation (Goumas et al., 2025). This can be problematic when analysing complex food matrices, potentially leading to variability or false responses if not properly controlled (Goumas et al., 2025). Despite these challenges, ongoing advances in surface engineering and material design continue to improve the stability, selectivity, and robustness of piezoelectric platforms (Pohanka, 2018).

## 20 Piezoelectric transducers for L. *monocytogenes*

Beyazit et al. (Beyazit et al., 2024) produced a QCM-based aptasensor combined with a magnetic pre-enrichment system for the detection of L. *monocytogenes*. Fe₃O₄ magnetic nanoparticles were coated with polydopamine (PDA) and dopamine-conjugated polyethylene glycol (DA-PEG) and then functionalised with an aptamer specific to L. *monocytogenes*. Fe₃O₄ (magnetite) nanoparticles are a ferrimagnetic nanomaterial with excellent conductivity, magnetic strength, and biocompatibility. This is due to its cubic inverse spinel structure, where magnetic iron cations (Fe^2+^ and Fe^3+^) occupy different crystalline positions that align to generate a strong net magnetic moment (Nguyen et al., 2021).

Meanwhile, PDA is formed under alkaline conditions (Tris-HCl, pH 8.5), allowing dopamine self-polymerisation to occur through oxidation. This creates a robust coating on the Fe₃O₄ nanoparticle surface. The interaction of PDA with Fe₃O₄ occurs through strong coordination interactions, including electrostatic and hydrogen bonding, ensuring stable attachment to the metal surface (Y. Li et al., 2018). PDA also acts as a universal primer, providing reactive functional groups for further functionalisation of the biosensing surface without the use of external activating agents (Taheri Ostad et al., 2026).

DA-PEG is then anchored to this surface through its amine groups interacting with PDA via a Schiff base reaction (Jung et al., 2018). DA-PEG exhibits strong antifouling, biocompatibility, and hydrophilicity properties, making it suitable for the pre-enrichment system. Its hydrophilic nature allows it to attract water molecules, forming a hydration layer that prevents non-specific adhesion of proteins and other interfering substances typically present in complex food matrices. This occurs through steric hindrance and repulsion effects from the hydrated PEG layer (Campuzano et al., 2019; Zhang, Hu, et al., 2025). Therefore, this allows the QCM sensor to function effectively as a mass-change detector later, whilst ensuring the selective presence of L. *monocytogenes* without interference from food debris.

Glutaraldehyde serves as the crosslinker by attaching the amino-modified aptamers to the amine groups on DA-PEG, forming a Fe₃O₄@PDA@DA-PEG-Apt complex. An external magnet is then applied, so the Fe₃O₄@PDA@DA-PEG-Apt particles are separated from the sample along with the bound L. *monocytogenes*. This system integrates magnetic separation, chemical functionalisation, and bioreceptor recognition into a single platform for the pre-concentration phase. The resulting complex, when used in complex food samples, allows efficient and selective binding of L. *monocytogenes*, removes impurities, and concentrates the bacteria prior to detection. Afterwards, a solution of NaOH combined with SDS is used to break the L. *monocytogenes*–aptamer interaction. NaOH increases the pH, weakening the non-covalent interactions whilst also altering the aptamer structure. Whereas SDS disrupts adsorption and assists with bacteria detachment. This results in the elution of L. *monocytogenes* into a clean, concentrated sample ready for biosensing detection.

The QCM biosensor is composed of a gold-coated quartz crystal modified with PDA and DA-PEG layers. The quartz crystal is held in place using O-rings, which secure the fragile crystal without damaging its vibration while also preventing liquid leakage from the flow cell system (Zafirah et al., 2021). Aptamers are also immobilised on this QCM surface, forming a Chip@PDA@DA-PEG-Apt biosensing interface. Once the sensing surface is prepared, the QCM crystal is exposed to a flow system containing the eluted L. *monocytogenes* sample from the pre-concentration step. This allows the aptamers on the sensing interface to bind specifically to L. *monocytogenes*. The flow-cell setup enables real-time monitoring of bacterial detection, as frequency changes can be observed immediately when the bacteria interact with the sensor surface. The detection mechanism occurs via mass change on the sensing surface, measured through shifts in resonance frequency. When L. *monocytogenes* binds to the sensing interface, the mass increases due to the size of the bacterial cells. This leads to slower crystal vibration and a decrease in frequency. Therefore, the frequency shift is proportional to the number of bacteria bound; as more bacteria attach, the mass increases, resulting in a larger decrease in frequency.

This QCM biosensor achieved a linear detection range from 1.0 × 10^2^ to 1.0 × 10^7^ CFU/mL and an LOD of 148 CFU/mL using the enriched sample fraction. The biosensor performed effectively in milk and chicken samples, with recovery rates between 82.5% and 91.8%. Strong specificity was also demonstrated, including discrimination against other *Listeria* species (*L. ivanovii, L. innocua, L. seeligeri*) and other bacteria such as *E. coli, S. aureus,* and *B. subtilis*. Therefore, this biosensor design illustrates an integrated approach that combines both pre-enrichment and detection systems into a single platform, whilst addressing real-life food matrix interference.

## 21 Piezoelectric transducers for *Salmonella*

A different QCM-based strategy was presented by Min et al. (Min et al., 2022) who developed an immunosensor using polyclonal antibodies to target *S. typhimurium*. Binding of the bacterial cells to the antibody-coated crystal surface produced measurable frequency shifts, though the authors noted that the initial shifts were relatively small. To amplify the response, AuNPs were introduced, which increased mass loading and enhanced the frequency change, allowing the sensor to reach a detection limit of 10^3^ CFU/mL. While the sensitivity was acceptable, the specificity was limited by the use of polyclonal antibodies. Significant cross-reactivity was observed, with non-*Salmonella* bacteria, such as *Listeria* producing similar responses. This highlights a recurring challenge in piezoelectric biosensing: unless highly selective bioreceptors are used, mass-based detection is vulnerable to false positives from non-target organisms with comparable size or surface properties.

## 22 Critical analysis

The use of a controlled flow-cell system in Beyazit et al.'s (Beyazit et al., 2024) study contributes significantly to real-time monitoring and system robustness, which are vital for real-life applications. The continuous flow process enables bacterial samples to pass across the biosensing surface in a controlled manner. When *L. monocytogenes* binds to the aptamers on the Chip@PDA@DA-PEG-Apt layer, the resulting frequency change is measured in real time, allowing continuous observation of bacterial binding events. The use of a flow-cell configuration is valuable as it improves reproducibility and better simulates real-world testing conditions (Zhang, Ma, et al., 2025).

However, mechanical and environmental parameters such as viscosity or temperature can influence the quartz crystal, causing variations in the measured frequency (Alexander et al., 2019). Although the study utilises a flow-cell system to minimise these effects, sensitivity challenges may still arise due to such factors affecting detection accuracy.

In the study by Beyazit et al. (Beyazit et al., 2024), the addition of a magnetic pre-enrichment step significantly enhances the efficiency of the detection system. Typically, complex food matrices cause signal interference due to components such as lipids and proteins adhering to the sensor surface (Sangubotla et al., 2026). However, the use of Fe₃O₄@PDA@DA-PEG-Apt particles mitigates these limitations through the combined properties of magnetite nanoparticles and antifouling polymer layers. The integration of PDA and DA-PEG provides strong antifouling, biocompatibility, and hydrophilicity (Campuzano et al., 2019; Zhang, Hu, et al., 2025). Thus, reducing non-specific adsorption of unwanted components. The study reports recovery efficiencies of up to 91.8%, demonstrating that the majority of L. *monocytogenes* cells are successfully captured prior to detection. This approach overcomes diffusion limitations, as the bacteria are concentrated into a smaller volume, increasing the number of cells delivered to the QCM surface and improving mass-based detection sensitivity.

However, while PEG is widely used for antifouling, it is not entirely effective under all conditions. In complex food matrices, PEG–protein interactions may still occur. At higher molecular weights or under certain conditions, PEG can exhibit amphiphilic behaviour, allowing proteins to interact via electrostatic forces or hydrogen bonding instead of being repelled (Wu et al., 2014). These interactions may induce conformational changes, leading to stronger attachment of proteins to PEG chains. Additionally, PEG can undergo oxidative degradation, reducing its antifouling effectiveness over time (D'Agata et al., 2021). This may result in increased background signals or potential false positives, particularly in complex food matrices containing diverse components.

Furthermore, achieving consistent antifouling layers during manufacturing may be challenging, which could impact reproducibility and scalability for industrial applications. This may require extensive validation and optimisation, potentially increasing cost and limiting large-scale implementation (Alexander et al., 2019).

Finally, the reported LOD of 148 CFU/mL in Beyazit et al.'s (Beyazit et al., 2024) study indicates a lower sensitivity compared to other biosensing strategies. Considering the strict detection requirements for L. *monocytogenes* in food safety regulations (e.g., absence in 25 g or ≤ 100 CFU/g), further optimisation would be necessary to improve sensitivity for practical food-industry applications (D'Ambrosio et al., 2026).

Overall, compared with electrochemical and optical biosensors, piezoelectric systems remain less extensively explored for the detection of L. *monocytogenes* and *Salmonella*. The limited number of available studies may reflect the technical challenges inherent in mass-based sensing, particularly the susceptibility of piezoelectric crystals to environmental and matrix-related interference. Nonetheless, the examples discussed demonstrate that QCM platforms are capable of reliable whole-cell detection and benefit from label-free real-time operation. Continued advancements in surface coatings, nanomaterial-assisted amplification, and selective bioreceptors such as aptamers are expected to enhance the practicality and sensitivity of piezoelectric biosensors, making them more competitive with electrochemical and optical modalities in future food safety applications.

## 23 Future perspectives

**Fig. 4:**
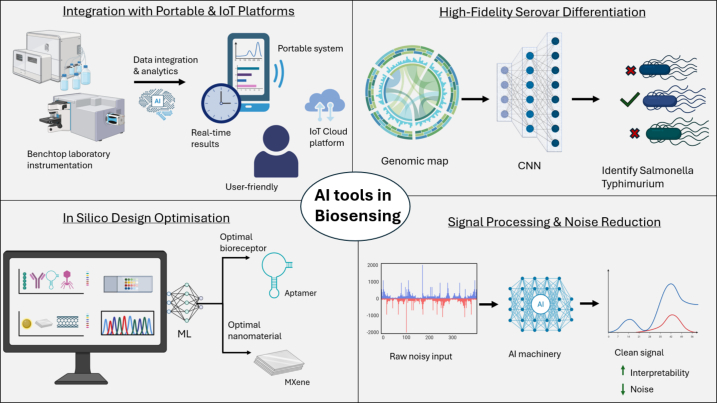
Applications of Artificial Intelligence (AI) across the biosensing workflow. The schematic overview highlights four key functional domains: (top left) the transition from bulky benchtop equipment to portable and IoT-integrated platforms for real-time results; (bottom left) in silico design optimisation for the predictive selection of optimal bioreceptors (e.g., aptamers) and nanomaterials (e.g., MXenes); (bottom right) signal processing strategies to reduce noise and improve data interpretability; and (top right) high-fidelity serovar differentiation using CNN-based identification of foodborne pathogen strains e.g. *Salmonella Typhimurium*. Created with BioRender.com.

The addition of artificial intelligence (AI) within biosensing is deeply shaping the development of biosensors for L. *monocytogenes* and *Salmonella* detection (Banicod et al., 2025; Engku Abd Rahman et al., 2026). As illustrated in Fig. 4, AI tools are being installed across four critical domains to overcome traditional analytical bottlenecks. Firstly, AI aids the in-silico design optimisation, allowing a predictive selection of the optimal nanomaterial and bioreceptor conjugates to improve binding affinity (Zhang, Tan, et al., 2025). Secondly, signal processing and noise reduction algorithms are used to unravel complex datasets, converting raw noisy data into clear indicative signals (Banicod et al., 2025; C. Zhang et al., 2024). Thirdly, high-fidelity serovar differentiation is achieved using Convolutional Neural Networks (CNNs), which accurately identifies specific pathogen strains from genomic data (Banicod et al., 2025; Hussain et al., 2022; Zhao et al., 2025). Finally, AI enables the integration of biosensors with portable and IoT (Internet of Things) platforms, transforming bulky benchtop laboratory instrumentation into real-time monitoring systems for enhanced food safety surveillance (Banicod et al., 2025).

AI can enhance bioreceptor selectivity by utilising machine learning (ML) strategies (Zhao et al., 2025). Such strategies can be used for screening high affinity ligands, which is specifically crucial for pathogens like L. *monocytogenes* and *Salmonella* that have closely related serovars (Banicod et al., 2025; Hussain et al., 2022; Zhao et al., 2025). Utilising this type of strategy is evident within studies such as those performed by Ding et al. (J. Ding et al., 2021) wherein AI-aided spectral processing allowed very similar strains of *Salmonella* to be distinguished successfully via multiscale convolutional neural networking (CNN), with AuNP-SERS spectral patterns achieving over 97% classification efficiency. A similar approach can, for instance, be implemented in the future for a dual-pathogen biosensor design targeting both L. *monocytogenes* and Sa*lmonella*.

Likewise, ML can be used to optimise transducer behaviour, as shown by Zhang, Tan, et al. (2025) where a bidirectional photoelectrochemical biosensor was constructed to detect *S. aureus*. In this study, ML tools optimised surface chemistry and guided the selection of nanomaterials that had minimal food matrix interferences. This is relevant for current *Listeria* and *Salmonella* sensing platforms, where majority depend on complex materials that improve sensitivity but compromise portability, manufacturability and overall cost (Banicod et al., 2025). Leveraging ML to assist with material modelling could allow researchers to select less expensive options more quickly by predicting the performance of piezoelectric, electrochemical and optical transducers using large datasets, thus reducing trial-and-error experimentations (Banicod et al., 2025; Goumas et al., 2025).

In addition to developing biosensors, there is strong potential for AI to enhance downstream signal interpretation, particularly considering problems associated with signal variability in food matrices (Banicod et al., 2025). The signals obtained from biosensors are frequently affected by factors such as noise, viscosity, temperature changes, and other matrix interactions, affecting precision when applied outside a controlled research setting (C. Zhang et al., 2024). AI-driven signal processing algorithms can address this by eliminating noise in real time whilst modifying operational parameters, improving sensor accuracy even in the presence of external variation (Deng et al., 2025). These approaches can also make the results more interpretable to non-technical user's by translating information obtained via impedance or optical signals into simple positive/negative results (Banicod et al., 2025; C. Zhang et al., 2024).

Notably, AI can also assist with biosensor miniaturisation. For example, Kang et al. (Kang et al., 2024) showed a compact handheld device that incorporate a Raman system together with CNN-based analysis to detect five foodborne pathogens, proving that high detection rates can also occur outside bulky spectroscopy equipment. The incorporation of such architectures into L. *monocytogenes* and *Salmonella* biosensors allows true on-field detection, which is one of the main hindrances discovered across various current biosensors (Banicod et al., 2025).

Future research should prioritise affordable, scalable biosensor designs that incorporate AI into user-friendly hardware such as smartphones (Kumar & Malviya, 2025; Pandhi et al., 2025). For instance, paper-based colorimetric biosensors with simple mobile AI applications could provide inexpensive screening options that avoid complex instrumentation altogether (Baştürk et al., 2024). Ultimately, the integration of AI tools for bioreceptor discovery, smart material design, automated signal correction as well as portable data processing will drive the development of the next generation *Salmonella* and L. *monocytogenes* biosensors (Banicod et al., 2025; Engku Abd Rahman et al., 2026). These future platforms will not only offer higher sensitivity and selectivity but will also be practical for field applications, easier to manufacture and suitable for routine use across the food industry.

## 24 Conclusion

Biosensor technology continues to show strong potential for improving the detection of L. *monocytogenes* and *Salmonella*, offering faster, more sensitive, and practical alternatives compared to traditional methods. The bioreceptors discussed, for instance, antibodies, aptamers, and bacteriophages demonstrate how recognition chemistry directly shapes sensor performance, with each offering its own strengths in specificity, robustness, or cost, but also challenges in stability, immobilisation, or real-sample compatibility. Likewise, the three major transducer platforms illustrate different capabilities: electrochemical sensors provide low-cost portability, optical systems achieve some of the lowest reported LODs (especially for *Listeria*), and piezoelectric devices enable whole-cell detection yet remain comparatively underexplored. While recent studies show remarkable progress, limitations such as matrix interference, surface chemistry optimisation, scalability, and dependence on complex instrumentation still restrict widespread use in food settings. Looking forward, the integration of artificial intelligence has the potential to address many of these issues through AI-guided bioreceptor screening, material design, automated signal correction, and sensor miniaturisation, ultimately enabling more reliable and user-friendly pathogen detection. Together, advances in bioreceptors, transducer engineering, and AI-assisted optimisation will shape the next generation of biosensors and help bridge the gap between laboratory performance and real-world application in the food industry.

## CRediT authorship contribution statement

**Suzan Efife Gumush:** Writing – review & editing, Writing – original draft, Investigation. **Rima Al-Attar:** Writing – review & editing, Writing – original draft, Investigation. **Ebba Sandbecker:** Writing – review & editing, Supervision. **Santosh Pandit:** Writing – review & editing, Supervision, Conceptualization.

## Declaration of competing interest

The authors declare that they have no known competing financial interests or personal relationships that could have appeared to influence the work reported in this paper.

## Data Availability

Data will be made available on request.
